# Immunohistochemical study of vascular endothelial growth factor-C/vascular endothelial growth factor receptor-3 expression in oral tongue squamous cell carcinoma: Correlation with the induction of lymphangiogenesis

**DOI:** 10.3892/ol.2015.3565

**Published:** 2015-08-04

**Authors:** TOMOFUMI NARUSE, SOUICHI YANAMOTO, SHIN-ICHI YAMADA, HIDENORI TAKAHASHI, YUKI MATSUSHITA, NAOMI IMAYAMA, HISAZUMI IKEDA, TAKESHI SHIRAISHI, SHUICHI FUJITA, TOHRU IKEDA, IZUMI ASAHINA, MASAHIRO UMEDA

**Affiliations:** 1 Department of Clinical Oral Oncology, Graduate School of Biomedical Sciences, Nagasaki University, Nagasaki, Nagasaki 852-8588, Japan; 2 Department of Regenerative Oral Surgery, Graduate School of Biomedical Sciences, Nagasaki University, Nagasaki, Nagasaki 852-8588, Japan; 3 Department of Oral Pathology and Bone Metabolism, Graduate School of Biomedical Sciences, Nagasaki University, Nagasaki, Nagasaki 852-8588, Japan

**Keywords:** tongue cancer, vascular endothelial growth factor-C, vascular endothelial growth factor receptor-3, podoplanin, regional recurrence

## Abstract

The aim of the present study was to elucidate the associations between the expression of the vascular endothelial growth factor-C (VEGF-C)/VEGF receptor-3 (VEGFR-3) axis and lymphangiogenesis, regional lymph node metastasis and clinicopathological factors in oral tongue squamous cell carcinoma (OTSCC) using immunohistochemistry. The expression of VEGF-C, VEGFR-3 and podoplanin was immunohistochemically evaluated in specimens obtained from 65 patients with OTSCC (T1-2, N0) who had undergone radical surgery alone. The associations between the expression of VEGF-C, VEGFR-3 and podoplanin, and lymphangiogenesis, regional lymph node metastasis and clinocopathological factors were determined by immunohistochemical analysis. VEGF-C, VEGFR-3 and combined VEGF-C/VEGFR-3 expression was significantly higher in cases with regional recurrence compared with those without lymph node involvement (P<0.001). As regards lymphangiogenesis, a significant correlation was observed between podoplanin expression and VEGF-C, VEGFR-3 and combined VEGF-C/VEGFR-3 expression (P<0.001). Therefore, lymphangiogenesis in the peritumoral stroma was associated with lymph node metastasis. However, podoplanin expression did not exhibit a significant correlation with the progression of lymph node metastasis. The results of the present study suggest that the VEGF-C/VEGFR-3 axis may be associated with lymph node metastasis through lymphangiogenesis. Determining the VEGF-C/VEGFR-3 expression status may help predict which patients will develop regional recurrence and provide novel targets for therapies to suppress lymph node metastasis in the treatment of OTSCC.

## Introduction

Oral tongue squamous cell carcinoma (OTSCC) accounts for ~1.5% of all cancer cases and is an aggressive cancer, frequently associated with a poor prognosis (1,2). The 5-year disease-specific survival (DSS) rate for patients with OTSCC has remained at 50–70% over the last 20 years (2–4). The current management and treatment of OTSCC for the majority of patients is surgery, with generally promising outcomes (4,5). In patients with early-stage OTSCC (T1-2, N0), the reported 5-year survival rates range between 75 and 89% (2,6,7). Interstitial brachytherapy is considered to be a viable treatment option for patients with early-stage OTSCC, since it results in a better functional preservation of swallowing and articulation compared with surgery and is associated with a satisfactory local control rate (8). However, certain patients with early-stage OTSCC may have a poor prognosis due to lymph node metastasis. Over 30% of patients with OTSCC exhibit cervical lymph node metastases, even in clinically node-negative disease. Among patients with early-stage OTSCC, the regional recurrence rate of the untreated neck is 20–30% (3,9). However, there remains controversy regarding the treatment of the clinically negative neck in such patients (10). Therefore, the role of the lymphatic system in OTSCC metastasis must be elucidated.

Although regional lymph node metastasis represents the first step of tumor dissemination for a variety of common types of human cancer, the molecular mechanisms underlying lymphatic metastasis are incompletely understood (11–13). Lymph node metastasis may be caused by the invasion of tumor cells in pre-existing lymphatic vessels in the tumor periphery, or through the induction of lymphangiogenesis via growth factor production (11,12,14). Lymphangiogenic growth factors produced by tumor cells and tumor-associated macrophages stimulate growth and dilation of the peritumoral lymphatic vessels and facilitate tumor cell entry through the lymphatic endothelium (11,13). Vascular endothelial growth factor-C (VEGF-C) is one of the lymphangiogenic growth factors of the platelet-derived growth factor/VEGF family. VEGF-C is an essential chemotactic and survival factor during embryonic and inflammatory lymphangiogenesis. VEGF-C is predominantly expressed along with VEGF receptor-3 (VEGFR-3), mainly in lymphatic capillaries, where it activates the development of tumor-associated lymphatic vessels and facilitates the access of tumor cells into these vessels (13–16). Several studies have reported that the VEGF-C/VEGFR-3 axis is associated with lymph node metastasis and that its expression is a prognostic factor for various cancers of the esophagus (17,18), stomach (19), colorectum (20), lung (21), cervix (22,23), prostate (24) and head and neck (25). Increasing evidence suggests that the VEGF-C/VEGFR-3 axis is associated with lymphangiogenesis, regional lymph node metastasis and poor prognosis. However, the role of the VEGF-C/VEGFR-3 axis in OTSCC has yet to be examined in detail. In the present study, immunohistochemical staining was used to investigate whether the expression of VEGF-C and VEGFR-3 is associated with lymphangiogenesis, regional lymph node metastasis and clinocopathological factors.

## Materials and methods

### 

#### Patients

The records of 65 patients who underwent radical surgery alone for early-stage (T1-2, N0) OTSCC between January, 2001 and December, 2011 were retrospectively reviewed. Paraffin-embedded sections of resected specimens were obtained (26). Tumor staging was performed according to the TNM classification of the International Union Against Cancer (27). Histological differentiation was defined according to the World Health Organization classification. The pattern of invasion (POI) was classified according to Bryne's classification (28).

All the study patients underwent extensive pretreatment evaluation, including physical examination, computed tomography (CT), magnetic resonance imaging, ultrasonography and positron emission tomography/CT for cervical lymph node metastasis. Patients diagnosed with no cervical lymph node involvement do not routinely undergo elective neck dissection; however, 4 patients in the present study had undergone neck dissection due to reconstruction, of whom 2 patients were found to have pathological occult cervical lymph node metastasis, which was defined as regional recurrence. Regional recurrence was defined as disease recurrence in the neck alone. Previous studies have demonstrated that the depth of invasion (DOI) is of predictive value for lymph node metastasis in patients with T1-2, N0 OTSCC (29–33). In the present study, DOI was classified into two groups, namely the ≥4 and <4 mm groups.

#### Immunohistochemical staining and evaluation

Sections deparaffinized in xylene were soaked in 10 mmol/l citrate buffer (pH 6.0) and placed in an autoclave at 121°C for 5 min for antigen retrieval. Endogenous peroxidase was blocked by incubation with 0.3% H_2_O_2_ in methanol for 30 min. Immunohistochemical staining was performed using the EnVision system (EnVision™+; Dako, Glostrup, Denmark). The following primary antibodies were used: Rabbit polyclonal antibodies against VEGF-C (cat. no. 18-2255; Invitrogen Life Technologies, Carlsbad, CA, USA; dilution, 1:100) and VEGFR-3 (cat. no. ab27278; Abcam, Cambridge, UK; dilution, 1:200). The sections were washed in Dulbecco's phosphate-buffered saline (PBS; Wako Pure Chemical Industries, Ltd., Osaka, Japan), followed by incubation with the primary antibodies at 4°C overnight. The reaction products were visualized by immersing the sections in diaminobenzidine solution and the samples were counterstained with Mayer's hematoxylin (Wako Pure Chemical Industries, Ltd.) and mounted.

VEGF-C protein expression was evaluated by calculating the total immunostaining score as the product of the proportional and the intensity scores. As previously described (25,34), proportional scores are based on the estimated fraction of positively-stained tumor cells (0, none; 1, <10%; 2, 10–50%; 3, 50–80%; and 4, >80%). The intensity score represents the estimated staining intensity (0, no staining; 1, weak; 2, moderate; and 3, strong). Therefore, the total immunostaining score ranges between 0 and 12. Positive cases were defined as those with a total score of >4, as the patient samples exhibited a bimodal distribution of immunohistochemical expression, where the discriminating nadir was a total score value of 3–4. VEGFR-3 staining was defined as positive if the vessel structures in the tumor stroma were highlighted. If no vessel structure was highlighted in the tumor stroma, the specimen was classified as negative. The evaluation of the combined VEGF-C/VEGFR-3 expression was classified into two categories: If the VEGF-C expression score was 0–3 and VEGFR-3 expression was negative, VEGF-C/VEGFR-3 expression was considered to be negative. If either the VEGF-C expression score was >4 or VEGFR-3 was positive, VEGF-C/VEGFR-3 expression was considered to be positive.

Lymphatic vessels were highlighted by staining lymphatic endothelial cells with mouse monoclonal antibody against podoplanin (cat. no. DK-2600; Dako; dilution, 1:100) and the number of lymphatic vessels was counted according to the hot-spot method (9,34). In brief, the area of highest lymphovascular density in the peritumoral stroma was identified by examining hematoxylin and eosin-stained sections under a Leica DM500 light microscope (Leica Microsystems, Inc.- Buffalo Grove, IL, USA; magnification, x200). The total number of lymphatic vessels in each of three visual fields was counted and the mean values were calculated. All immunohistochemical assessments were performed by two examiners in a blinded manner.

#### Statistical analysis

The associations between the expression of target molecules and clinicopathological characteristics were analyzed by the Fisher's exact test. Continuous data are presented as means ± standard deviation. The DSS rate was calculated by the Kaplan-Meier method and compared using the log-rank test. A multiple regression study was performed using Cox proportional hazards analysis. Predictors that were not associated with DSS or lymph node metastasis were not included in the multivariate analysis. The difference in podoplanin expression between the groups was evaluated by the Student's t-test. P<0.05 was considered to indicate a statistically significant difference.

## Results

### 

#### Patient characteristics

The clinicopathological characteristics of the patients are summarized in Table I. Between 2001 and 2011, a total of 65 patients were evaluated 53.8% of whom were male and 46.2% female. The mean age of the patients was 64.2 years (range, 28–86 years). The regional recurrence rate was 20% (13/65 patients) during the follow-up period; of these, 8 cases were pN1 and 5 were pN2. Of the 65 cases, 7 were extracapsular spread (ECS)-positive.

#### Expression of VEGF-C, VEGFR-3 and podoplanin in OTSCC

**Figure 1.**
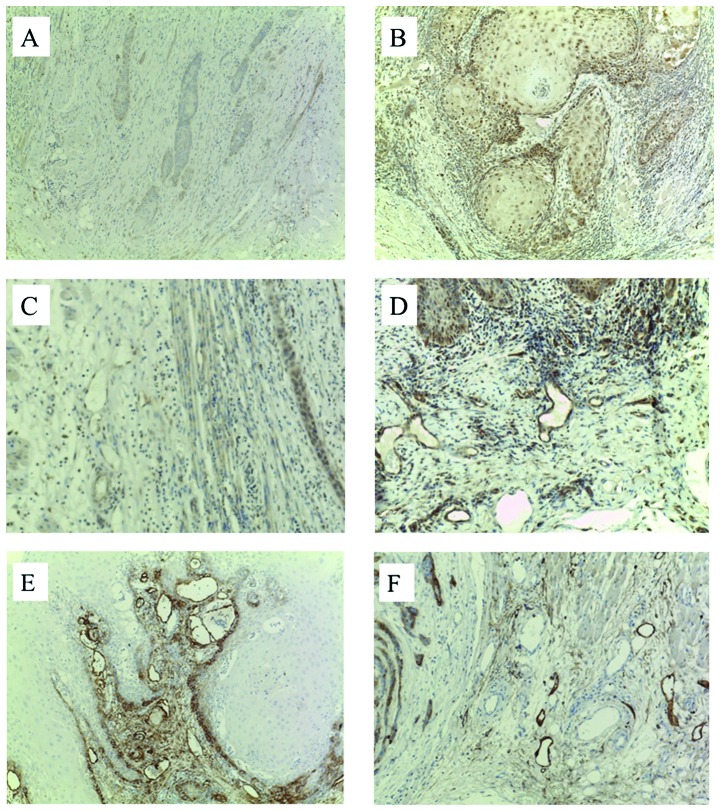
Representative immunohistochemical staining for VEGF-C, VEGFR-3 and podoplanin. (A) Negative staining for VEGF-C in OTSCC. (B) OTSCC with Bryne's score 2, displaying strong VEGF-C cytoplasmic expression (staining index, 12). Negative staining for VEGFR-3 (C) in the peritumoral stroma and (D) at the invasive front. Podoplanin expression (E) in the peritumoral stroma and (F) at the invasive front. VEGF-C, vascular endothelial growth factor-C; VEGFR-3, vascular endothelial growth factor receptor-3; OTSCC, oral tongue squamous cell carcinoma.

Among the 65 patients with OTSCC, the immunohistochemical staining was positive for VEGF-C in 78.4% and for VEGFR-3 in 55.4% of the samples. VEGF-C was expressed primarily in the cytoplasm of the tumor cells, with the intensity ranging between weak and strong. The distribution of VEGF-C was observed in tumor nests and at the invasive front, with particularly strong expression observed at the invasive front (Fig. 1A and B). In the peritumoral stroma, VEGFR-3-positive small-diameter lymphatic vessels were observed (Fig. 1C and D). Podoplanin was expressed in the microvascular structures and the cytoplasm of tumor cells, with podoplanin-positive microvascular structures clearly detected in the peritumoral stroma and connective tissue. Podoplanin expression was absent in tumor nests and was found only in the basal cell layer, with diffuse and extensive expression in the cytoplasm of the tumor cells (Fig. 1E and F).

#### Associations of VEGF-C and VEGFR-3 expression with clinicopathological characteristics and survival

VEGF-C and VEGFR-3 expression in OTSCC was investigated as a function of clinicopathological characteristics. VEGF-C expression was significantly associated with clinical growth pattern (P<0.01) and DOI (P<0.05). There was no significant association between VEGF-C expression and POI or regional recurrence. VEGFR-3 expression was significantly associated with clinical growth pattern, POI, DOI and regional recurrence (P<0.01, <0.05, <0.01 and <0.05, respectively) (Table II). Combined VEGF-C/VEGFR-3 expression was significantly associated with clinical growth pattern, DOI and regional recurrence (P<0.001, <0.001 and <0.05, respectively) (Table III).

**Figure 2.**
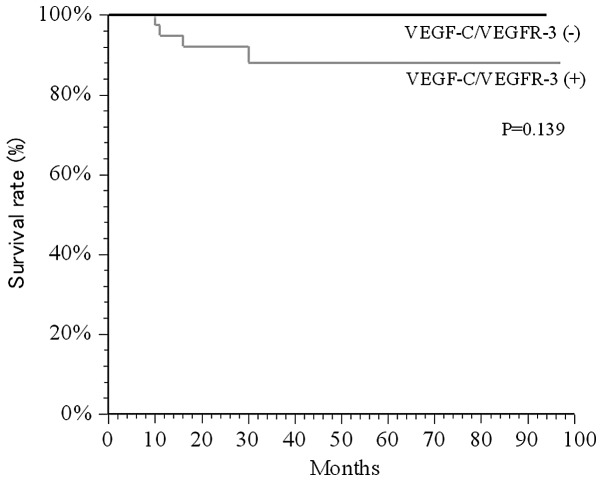
Kaplan-Meier survival curve of the 5-year DSS rate. No significant correlation between VEGF-C/VEGFR-3-positive and -negative patients was observed regarding the 5-year DSS rate (P=0.139). Differences between the two groups were evaluated by the log-rank test. DSS, disease-specific survival; VEGF-C, vascular endothelial growth factor-C; VEGFR-3, vascular endothelial growth factor receptor-3.

The 5-year DSS rate according to the combined VEGF-C/VEGFR-3 expression was determined. The univariate analysis using the log-rank test and the Kaplan-Meier method demonstrated that combined VEGF-C/VEGFR-3 expression was likely to be associated with 5-year DSS, but no significant difference was observed between positive and negative cases (Fig. 2, P=0.139).

#### Association of VEGF-C and VEGFR-3 expression with regional recurrence

The univariate logistic analysis revealed a significant association of regional recurrence with T stage (P=0.03), clinical growth pattern (P<0.01), POI (P<0.001), DOI (P<0.001) and VEGF-C/VEGFR-3 (P=0.043) (Table IV). A multivariate logistic regression analysis was performed for each predictor of regional recurrence, and VEGF-C/VEGFR-3 was not identified as an independent factor for regional recurrence (Table V).

#### Association of lymphatic vessel count with the expression of VEGF-C/VEGFR-3 and the progression of pathological lymph node metastasis

A significant association was observed between combined VEGF-C/VEGFR-3 expression and lymphatic vessel count (P<0.001). Furthermore, when VEGF-C/VEGFR-3-positive and -negative cases were compared, the lymphatic vessel count was significantly higher in VEGF-C/VEGFR-3-positive cases (P<0.001). Lymph node metastasis was subdivided into two groups: 13 cases were classified as pN0 and 52 cases as pN1–2. The mean lymphatic vessel count was significantly higher in the pN-positive group compared with that in the pN-negative group (P<0.0001). For further analysis, the pN-positive group was subdivided into four categories, namely pN1, pN2, ECS-negative and ECS-positive, in order to investigate the correlation between the progression of lymph node metastasis and lymphangiogenesis. However, no significant correlation was observed (Table VI).

## Discussion

Further insight regarding VEGF-C/VEGFR-3 expression in OTSCC may improve our understanding of lymphangiogenesis and regional lymph node metastasis, in addition to providing novel treatment possibilities for OTSCC. Therefore, the present study aimed to determine the expression of molecules associated with lymphangiogenesis, lymph node metastasis and survival rate in OTSCC.

VEGF-C expression was found to be associated with clinical growth pattern and DOI, whereas the expression of VEGFR-3 was associated with POI, clinical growth pattern, regional recurrence and DOI. The expression of the VEGF-C/VEGFR-3 axis was found to be associated with clinical growth pattern, regional recurrence and DOI, but not with 5-year DSS. Furthermore, the expression of the VEGF-C/VEGFR-3 axis was found to be predictive of regional recurrence (odds ratio = 8.8), T stage (T2), diffuse POI, endophytic growth pattern and DOI (≥4 mm) in the univariate analysis. These results suggest that the VEGF-C/VEGFR-3 axis may be associated with the mechanism of regional recurrence in OTSCC.

Previous studies denonstrated that VEGF-C and VEGFR-3 expression is significantly associated with lymph node metastasis in esophageal SCC, gastric, cervical and head and neck cancer (17–19,22,25). Furthermore, VEGF-C and VEGFR-3 expression was found to be significantly associated with poor survival rates in patients with esophageal SCC and gastric carcinoma (18,19). However, two previous studies reported no association between VEGF-C and VEGFR-3 expression and lymph node metastasis in lung and colorectal cancer (20,21). These differences in study findings may be due to the different anatomical locations of the tumors, differences in the study method, or differences in the cut-off value for positivity. However, high-intensity expression was observed at the invasive front in the present study, indicating that VEGF-C and VEGFR-3 play a major role in lymph node metastasis in OTSCC. The significant correlation between the expression of the VEGF-C/VEGFR-3 axis and 5-year DSS may be attributed to the present study focusing on early-stage OTSCC.

It has been reported that lymphangiogenesis is associated with lymph node metastasis, as peritumoral lymphatics located immediately adjacent to tumors or in the peritumoral stroma may be dilated or enlarged (11–13). A number of previous studies have used podoplanin or other lymphatic markers to verify this association (9,13,18,25,35,36). However, it has been reported that intratumoral lymphatic vessels may be poorly functional and not required for lymph node metastasis, as tumor cells may spread via pre-existing lymphatic vessels (11–13,37). Podoplanin, a mucin-like transmembrane glycoprotein, is one of the specific markers of lymph vessel endothelial cells. Podoplanin is highly and specifically expressed in the endothelial cells of lymphatic vessels, but not in those of blood vessels (36). Although its biological function is not yet clearly understood, a number of previous reports have suggested that podoplanin may act as a mediator of tumor cell invasion and metastasis (9,13,18,25,35,37). However, a previous study reported no significant correlation between podoplanin expression and tumor metastasis (37). Evidence regarding podoplanin expression in OTSCC, in particular, has demonstrated that the immunoreactivity to podoplanin is 97% and its expression exerts no effect on T stage (38). Therefore, the present study investigated the association of podoplanin with the VEGF-C/VEGFR-3 axia and the progression of lymph node metastasis.

The present study showed that the expression of podoplanin was clearly distributed in the peritumoral stroma and cytoplasm of tumor cells. The number of lymphatic vessels highlighted by podoplanin expression was associated with lymph node metastasis. Furthermore, the presence of VEGF-C, VEGFR-3 and the VEGF-C/VEGFR-3 axis was associated with an increased lymphatic vessel count, but not with the progression of lymph node metastasis. These results suggested that peritumoral lymphatic vessels may be functional and that lymphangiogenesis is associated with lymph node metastasis. It was previously demonstrated that podoplanin expression predicts the progression of lymph node metastasis (37). However, in the present study, no significant association with the progression of lymph node metastasis was observed. From these results, it may be hypothesized that podoplanin is a useful marker for predicting lymph node metastasis, but is of less value for predicting the progression of lymph node metastasis.

The hot-spot method is frequently used to count the lymphatic vessels highlighted by podoplanin expression (9,18,25,35,37. It was recently reported that podoplanin expression in tumor cells is associated with tumorigenesis in OSCC (36). Therefore, further investigation is required to determine the utility of evaluating podoplanin expression in patients with OTSCC.

The present study revealed that the VEGF-C/VEGFR-3 pathway is associated with lymph node metastasis through lymphangiogenesis. A potential limitation of the present study is its retrospective design. This type of study may be associated with inherent bias. In our department, resectable OTSCC is currently treated with surgery and/or adjuvant chemoradiotherapy to reduce the risk of recurrence. Patients who required neoadjuvant radiotherapy and/or chemotherapy, and who exhibited more advanced, unresectable disease, were excluded from the present study. Despite unifying the surgical modality, residual confounding may have occurred. Therefore, prospective study design is required to increase the evidence of the present study. Finally, since the incidence of OTSCC is low compared with other types of cancer, the present study included a small number of cases. Therefore, further intergroup studies are required to confirm our results. In conclusion, although the association between lymphangiogenesis and lymph node metastasis is controversial, determining the status of VEGF-C/VEGFR-3 expression may help predict which patients are at risk of developing regional recurrence and provide a novel target for the treatment of OTSCC through the suppression of lymph node metastasis.

## Figures and Tables

**Table I.** Clinicopathological characteristics of patients with oral tongue squamous cell carcinoma (n=65).

Characteristics	No. (%)
Age (years)	
Range	28–86
Mean	64.2
Gender	
Male	35 (53.8)
Female	30 (46.2)
T stage	
T1	38 (58.5)
T2	27 (41.5)
Clinical growth pattern	
Superficial	27 (41.5)
Exophytic	12 (18.5)
Endophytic	26 (40.0)
Histological differentiation	
High	62 (95.4)
Moderate	2 (3.1)
Poor	1 (1.5)
Depth of invasion (mm)	
<4	42 (64.6)
≥4	23 (35.4)
Pattern of invasion	
Grade 1	7 (10.8)
Grade 2	49 (75.4)
Grade 3	8 (12.3)
Grade 4	1 (1.5)
Neck treatment	
Observation	61 (93.8)
Dissection	4 (6.2)
Regional recurrence	
No	52 (80.0)
Yes	13 (20.0)
pN classification	
pN1	8 (61.5)
pN2	5 (38.5)
Extracapsular spread	
Negative	6 (46.2)
Positive	7 (53.8)

**Table II.** Associations of VEGF-C and VEGFR-3 expression with clinicopathological factors.

	VEGF-C expression			VEGFR-3 expression		
				
Characteristics	–	+	P-value	–	+	P-value
Age (years)			0.257			0.209
>65	5	24		10	19	
≤65	11	25		19	17	
Gender			0.5660			1.000
Male	0	25		16	19	
Female	6	24		13	17	
T stage			0.152			0.324
T1 + T2	12	26		19	19	
T3 + T4	4	23		10	17	
Differentiation			1.00			1.000
High	16	46		28	28	
Moderate/poor	0	3		1	8	
Pattern of invasion			0.569			0.036
Grades 1/2	15	41		28	28	
Grades 3/4	1	8		1	8	
Clinical growth pattern			0.0012			0.0056
Superficial + exophytic	15	24		23	16	
Endophytic	1	25		6	20	
Regional recurrence			0.159			0.027
No	15	37		27	25	
Yes	1	12		2	11	
Depth of invasion (mm)			0.0052			0.0088
<4	15	27		24	18	
≥4	1	22		5	18	

VEGF-C, vascular endothelial growth factor-C; VEGFR-3, vascular endothelial growth factor receptor-3.

**Table III.** Associations of VEGF-C/VEGFR-3 expression with clinicopathological factors.

	VEGF-C/VEGFR-3		
		
Characteristics	–	+	P-value
Age (years)			0.605
>65	9	20	
≤65	14	22	
Gender			0.799
Male	13	22	
Female	10	20	
T stage			0.408
T1 + T2	17	21	
T3 + T4	6	21	
Differentiation			0.546
High	23	39	
Moderate/poor	0	3	
Pattern of invasion			0.142
Grades 1/2	22	34	
Grades 3/4	1	8	
Clinical growth pattern			0.0001
Superficial + exophytic	21	18	
Endophytic	2	24	
Regional recurrence			0.0235
No	22	30	
Yes	1	12	
Depth of invasion (mm)			0.0009
<4	21	21	
≥4	2	21	

VEGF-C, vascular endothelial growth factor-C; VEGFR-3, vascular endothelial growth factor receptor-3.

**Table IV.** Clinicopathological characteristics and VEGF-C/VEGFR-3 expression in association with regional recurrence.

	Regional recurrence				
				
Characteristics	–	+	Odds ratio	95% CI	P-value
Age (years)			0.628	0.185–2.13	0.456
>65	22	7			
≤65	30	6			
Gender			2.250	0.615–8.23	0.221
Male	26	9			
Female	26	4			
T stage			4.250	1.148–15.73	0.031
T1	34	4			
T2	18	9			
Differentiation			9.270	0.770–111.54	0.079
High	51	11			
Moderate/poor	1	2			
Pattern of invasion			81.600	8.400–791.90	<0.001
Grades 1/2	47	5			
Grades 3/4	5	8			
Clinical growth pattern			32.570	3.800–274.10	<0.010
Superficial + exophytic	38	1			
Endophytic	14	12			
Depth of invasion (mm)			44.700	5.200–382.40	<0.001
<4	41	1			
≥4	11	12			
VEGF-C/VEGFR-3			8.800	1.060–72.79	0.024
Negative	22	1			
Positive	30	12			

VEGF-C, vascular endothelial growth factor-C; VEGFR-3, vascular endothelial growth factor receptor-3; CI, confidence interval.

**Table V.** Multivariate analysis in relation to regional recurrence.

Parameters	Odds ratio	95% CI	P-value
T stage (T1 + T2 vs. T3 + T4)	2.773	0.051–282.500	0.462
Pattern of invasion (grades 1/2 vs. 3/4)	34.745	1.990–605.670	0.015
Clinical growth pattern (Superficial + exophytic vs. endophytic)	2.202	0.150–31.050	0.559
Depth of invasion (<4 vs. ≥4 mm)	13.130	0.850–202.688	0.065
VEGF-C/VEGFR-3 (negative vs. positive)	3.787	0.050–282.500	0.545

VEGF-C, vascular endothelial growth factor-C; VEGFR-3, vascular endothelial growth factor receptor-3; CI, confidence interval.

**Table VI.** Associations between lymphatic vessel counts and the progression of lymph node metastasis and protein expression of VEGF-C/VEGFR-3.

Parameters	Mean lymphatic vessel counts^a^	P-value
VEGF-C		<0.0001
Negative	8.571±8.182	
Positive	17.176±8.757	
VEGFR-3		<0.0001
Negative	10.379±7.439	
Positive	19.306±8.784	
VEGF-C/VEGFR-3		<0.0001
Negative	9.217±7.552	
Positive	18.666±9.333	
pN classification		<0.0001
pN0	13.166±8.659	
pN1/2	25.727±5.515	
pN classification		0.099
pN1	29.500±6.908	
pN2	23.600±2.702	
Extracapsular spread		0.146
Negative	30±7.127	
Positive	22.5±4.670	

a Data are presented as means ± standard deviation. VEGF-C, vascular endothelial growth factor-C; VEGFR, vascular endothelial growth factor receptor-3.
